# Unidimensional scales for fears of cancer recurrence and their psychometric properties: the FCR4 and FCR7

**DOI:** 10.1186/s12955-018-0850-x

**Published:** 2018-02-09

**Authors:** G. M. Humphris, E. Watson, M. Sharpe, G. Ozakinci

**Affiliations:** 1 School of Medicine, University of St Andrews, St Andrews, Fife, Scotland; 2 Edinburgh Cancer Centre, Western General Hospital, Edinburgh, Scotland; 3 Department of Psychiatry, Warneford Hospital, Oxford, OX3 7JX England

## Abstract

**Background:**

The assessment of fear of recurrence (FCR) is crucial for understanding an important psychological state in patients diagnosed and treated for cancer. The study aim was to determine psychometric details of a seven question self-report scale (FCR7) and a short form (FCR4) based upon items already used in various extensive measures of FCR.

**Methods:**

Two consecutive samples of patients (breast and colorectal) were recruited from a single specialist cancer centre. The survey instrument contained the FCR7 items, Hospital Anxiety and Depression Scale (HADS), and demographic details. Clinical information was obtained from patient hospital records. Statistical analyses were performed using classical test and item response theory approaches, to demonstrate unidimensional factor structure and testing key parameters. Construct validity was inspected through nomological and theoretical prediction.

**Results:**

Internal consistency was demonstrated by alpha coefficients (FCR4: 0.93 and FCR7: 0.92). Both scales (FCR7 & FCR4) were associated with the HADs subscales as predicted. Patients who experienced chemotherapy, minor aches/pains, thought avoidance of cancer and high cancer risk belief were more fearful. Detailed inspection of item responses profile provided some support for measurement properties of scales.

**Conclusion:**

The internal consistency, and pattern of key associations and discriminability indices provided positive psychometric evidence for these scales. The brief measures of FCR may be considered for audit, screening or routine use in clinical service and research investigations.

**Electronic supplementary material:**

The online version of this article (10.1186/s12955-018-0850-x) contains supplementary material, which is available to authorized users.

## Background

Fears of cancer recurrence (FCR), has recently been defined by an expert panel in a three-wave Delphi study, as ‘fear, worry, or concern relating to the possibility that cancer will come back or progress ‘. There is substantial variation in the experience of this fear with a small percentage of patients who report no fears of this nature at all to those who state a specific concern of the cancer returning requiring specialist intervention [1].

Numerous attempts have been made to design measures of FCR and a review has listed those questionnaires. Authors of the review recommend further research to assist this process of development and refinement [2]. Thewes et al. argued that the lack of an accepted definition of FCR had resulted in many measures being developed. Simonelli et al. [3] has highlighted a number of issues requiring attention: 1) single point designs, 2) over-reliance on breast cancer survivors, 3) lack of cut-off point, 4) lack of conceptualisation of FCR, and 5) cross-cultural validity.

The assessment of FCR has taken a number of approaches [2]. They can be divided into two main types, including single item or multi-item scales. A single item scale is easy to administer, can be applied on multiple occasions, but psychometrically can be limited in measurement performance when investigators have attempted to determine some key parameters (e.g. reliability). For instance, single-item assessments of depression have been shown to have some serious shortcomings [4]. Multi-item scales have the benefit of assessing a variety of qualities or features of FCR [5]. However, they can be burdensome to complete, time-consuming and challenging to score and interpret. Costa et al. makes the case that FCR is best assessed using a single dimensional design consisting of a small set of items [6]. The rationale we believe that is being supported by Costa et al. is to identify the key area to be accurately measured and then follow up through further assessment with associated features. These areas can be assessed through, either specific packets of items in a further questionnaire, or a structured clinical interview. The short uni-dimensional measure can then be considered initially as a screening instrument for additional follow-up and enquiry.

The Concerns About Recurrence Questionnaire (CAR-Q) has been investigated as a possible brief scale for FCR [7]. It has been constructed as a 5 item scale that questions the respondent about frequency, intrusiveness and degrees of distress. An item of risk perception is also included. There is a translation into Danish as well as English therefore promoting a cross-cultural dimension. The authors used classical test theory (CTT) in addition to single parameter models (Item Response Models, IRT) to estimate difficulty (referred to also as ‘location’ or ‘severity’ across the latent construct). The CAR-Q was developed specifically for breast cancer. Our group has considered bringing together a scale that will function well across numerous cancer sites. This would save substantial effort and also enable comparisons across sites. The CAR-Q is perhaps the closest to what we would regard as a scale that has been studied in some detail psychometrically. There are some satisfactory features of this scale and the work by the originators is carefully reported. Their IRT results on further inspection do show some extensive local dependence issues which they do state in their 2015 article will need addressing and some items rewording. They state that CAR-Q-4 (the 4 item version) perhaps is the most reliable but admit that the 4 item English version did not fit the Rasch model and did not report the fit for the Danish sample saying that ‘modelling’ was not possible with the 4-item scale. Was this a convergence problem? This is not elucidated. The modelling also was rejected for this Danish 3 item version. Our interpretation suggests that there are some unsatisfactory psychometric qualities to this scale. Furthermore, no brief FCR scale has adopted the more flexible two parameter graded response model IRT approach. The advantage of this later approach is that both item discrimination (slope) as well as difficulty/severity (location) are estimated simultaneously.

The scale that we have developed and employed in a variety of centres in the UK (over 70 NHS units) [8] is based upon a set of 7 questions that have been selected from extant measures within the literature to assess directly FCR. The FCR4 contains the first four items of the FCR7. That is the FCR7 is the FCR4 with the addition of 3 additional items. The Flesch readability index for the FCR7 is 60.2 and the shorter 4 item version (FCR4) is 55.1, equivalent to ‘Plain English, easily understood by 13- to 15-year-old students’. Although used relatively extensively in the UK there are important considerations about the multi-item measure and its psychometric characteristics.

This paper reports on the evaluation of a 7 item FCR scale (FCR7) and that has already been presented in a sample of head and neck cancer patients in the North-west of England [9]. Our belief is that the item wording taken from many of the available measures would make suitable short screening instruments for routine clinical service use. Our rationale was to produce a patient acceptable and psychometrically sound short FCR scale. The four item version (FCR4) for example has been used in a major study with patients with head and neck cancer in the UK [10] and percentile norms have been presented (with confidence intervals) for clinicians to include in the patient records. The novel aspect of this paper includes the evidence obtained from CTT and IRT analytical approaches to both the four and seven item forms.

### Aim

To present two versions (4 and 7 items) of screening assessments for FCR and describe their psychometric features. The objectives are:

1. To present the selection of items comprising the measures and item wording;
2. To outline psychometric features based upon Classical Test Theory (CTT);
3. To summarize Item Response Theory (IRT) estimates for scale items;
4. To present recommendations for version choice and future research.

## Methods

### Sample

The setting for the study was the Edinburgh Cancer Centre where a tertiary regional service is provided with full radiotherapy, chemotherapy and surgery treatment modalities available. The Breast Cancer and Colorectal Units and their Multi-Disciplinary Teams gave approval for recruiting their patients. The participants consisted of consecutive patients attending the out-patient clinics of the two Units. Exclusion criteria were: serious mental illness, non-English speaking, treatment for recurrent cancer and terminally ill. During the data collection of 5 months there were 482 breast and 116 colorectal cancer patients available for recruitment. As some patients were undergoing further tests, the total eligible for written contact was 324 and 101 respectively which enabled a total pool of 425 patients (71% of 598).

### Measures

The FCR scale was based on examining past measures of this construct with a focus on anxiety or fear. Sixteen items were considered as candidates for the short versions. Through a consensus approach of cycling through these items between the authors and also piloting them with patients at the ECC to examine reactions to item wording we selected the final item pool. The four question scale (FCR4) was designed to feature anxiety, worry and strong feelings coupled with return of the disease (Q1–4). The longer seven question scale, to some extent was a compromise somewhat extending the item pool by featuring two items on the cognitive processing component that anxiety, fears or worries may interfere or potentially distort thoughts about cancer returning, Q5 and Q7. Finally, an item describing a behavioural response to FCR was included, Q6. Our approach for the FCR7 was to introduce additional items that were not simply variations of the first 4 items comprising the FCR4. One of the issues identified by patients when completing original formulations was the repetitive nature of some of the wording employed. Hence we introduced items that were worded with additional content (cognitive and behavioural) but possibly with substantial association with the core uni-dimensional construct. See Additional file 1: FCR7 for copy of Scale. The empirical data generated would enable inspection of the veracity of this approach.

To assist with validating the FCR measures (FCR4 and FCR7) a number of additional hypothetical constructs were assessed and their relationships tested using the construct validation approach outlined by Streiner and Norman [11]. This approach subsumes other forms of validity [12]. The Hospital Anxiety and Depression Scale (HADS) was administered [13]. We predicted that the FCR scales will be significantly related to anxiety and somewhat less so with depression, based upon a prior review [1], and adopting the two-factor model of the HADS measure [14]. In addition, a short number of single question items, were also included to assess the relationship with the total FCR scores. We included three items with 5 category ordinal rating scales ranging from ‘not at all’ to ‘all the time’. We deliberately chose statements that reflected some key areas known to be associated according to the three major components of Lee-Jones et al. model [15] of FCR derived from Leventhal’s self-regulation model (SRM), namely: representation of symptoms (‘Minor aches and pains remind me of cancer’), coping strategies (‘I just try to ignore feelings about the cancer returning’), and appraisal (‘In your opinion, what is your risk of having cancer recurrence?’). We expected the FCR scales to be positively associated with these components. We recognised that multi-item-scales are preferable to single items for assessing each of these components. However the inclusion of these clear statements reflecting the SRM would enable supportive evidence to be compiled. For example, a hypothesis was made that the avoidance coping strategy may not be as closely related to FCR as respondents may defer the process of accessing either, their fears, or admission of using avoidance [16, 17].

Finally, patients who undergo mixed treatment protocols, and therefore believe their condition to be more complex and difficult to irradicate would tend to have raised FCR levels [1]. Recently, this has been confirmed in meta-analyses conducted by our group [18, 19].

### Statistical analysis

Data were analysed using SPSS v22 (IBM Corp.) for descriptive analyses, and reliability analysis conducted with FACTOR [20]. Initially, the scales were factor analysed (principal factor method) [21] and Horn’s parallel analysis was run to determine the factorial structure [22]. Relationships between the total scores (FCR7 and FCR4) obtained from summing the items (without factor weights) were examined visually through scattergrams, polynomial smoothing graphics (STATA v14, StatCorp, College Station, Texas), and Pearson correlation coefficients. Spearman’s rho rank correlations were employed with the single question rating scale items to test association with the FCR4 and FCR7 scales. Alpha was set to 0.05 (two-sided) throughout.

The Item Response Theory (IRT) ‘grm’ procedure within STATA v14 was applied. This enables discriminability and difficulty parameters using maximum likelihood estimation to be produced simultaneously and graphically presented. The approach provides a much more intensive inspection of the performance of the FCR4 and FCR7 items than the more conventional CTT approach. The two assumptions are made when using IRT are unidimensionality and local item independence. The first assumption is easily tested by exploratory factor analysis and the examination of eigenvalues to assess the fit of a single latent construct of FCR. The second assumption is inferred from examination of the item wording.

### Ethical approval

The study was introduced to potential participants through a participant information sheet (PIS) and written consent obtained. A research assistant identified potential participants by reading through notes of patients planned for BT/CRC clinic visits. A letter then was sent out signed by their clinician inviting them to participate in the study, including a patient information sheet. The potential participant was met by the research assistant at clinic appointment, and they were given the opportunity to ask questions, and if they agreed to participate, they signed a consent form and answered the questionnaire. Patients were able to withdraw at any time, according to conventional ethical guidelines. The study was approved by NRES (07/S1103/27) and NHS Lothian R&D.

## Results

Persons who had received treatment at the tertiary cancer centre with disease of the breast (*N* = 206; mean age = 63) or colorectal cancer (*N* = 53 mean age = 67) attended the nurse-led follow-up clinics to complete questionnaire. Hence, from the total pool of eligible patients available we attained a 61% response rate (259/425). The frequency breakdown of demographics, cancer severity and details of tumour (node involvement and margins) are presented (Table 1). The majority of patients were females (226 vs 33) although within the colorectal group 60% were men. There was reasonable representation across age range. Patients aged 35 to 49 years were relatively few 21/259 (8%). About 10% of the sample were single (26/259) and 61% married (157/259). Educational level was mixed and the large majority (79%) were resident in owner-occupied accommodation. The cancer staging between sites was very different. Patients were characterised by low severity in breast cancer and advanced in colorectal cancer. Node involvement was comparable at approximately 2/3rds across sites. Margins tended to be clear. Patients with colorectal cancer, however had margins that were difficult to establish.

**Table 1:** Demographics of breast and colorectal samples

	Cancer Site			
	Breast (*N* = 206*)		Colorectal (*N* = 53*)	
Gender	N	%**	N	%**
Female	205	99	21	40
Male	1	1	32	60
Age				
35 to 49	19	9	2	4
50 to 59	57	28	13	25
60 to 69	84	41	17	32
70+	46	22	21	40
Marital Status				
Married	121	59	36	68
Divorced	19	9	5	9
Widowed	34	17	6	11
With partner	6	3	2	4
Single	22	11	4	8
Education				
No formal qualification	64	31	20	20
GCSE/O Levels	46	22	10	19
A Levels/Equivalent	38	18	4	8
Univ Degree	45	22	14	26
Higher Univ Degree	9	4	5	9
Accommodation				
Owner Occupier	164	80	40	76
Rented/Council	27	13	10	19
Private landlord	6	3	0	0
Other	4	2	2	4
Cancer Staging^a^				
I	51	54	3	6
II	33	35	5	10
III	6	6	33	67
IV	3	3	8	16
Node Involvement				
Yes	49	25	16	32
No	130	66	34	68
Unknown	17	9	0	0
Cancer Margins				
Clear	105	54	16	32
Not Clear	15	8	3	6
Unknown	76	39	31	62

*some missing participant responses as indicated; a. Missing data due to staging not available

**percentages rounded to integer level, therefore sum not always 100%

The responses to the FCR4 (and FCR7) questions are summarised across cancer sites as mean levels for individual items and total scores. The 60th and 90th percentile values for both scales across cancer sites are also presented (Table 2). Discussions had been conducted informally with clinicians (oncologists, surgeons and specialist nursing staff) who gave opinions seeking identification of their patients who scored at a moderately high level which was determined to be greater than the average. Hence a 60th percentile was regarded as a pragmatic level to report. In addition those clinicians who requested an estimated level of high FCR stated that the top one in ten scorers would be helpful to identify. Therefore pragmatic considerations that clinicians favoured drove our choice of percentile. Another approach was to produce detailed tables of deciles that may produce extensive but overwhelming information for the non-specialist psychometrician. This recruitment level for patients with colorectal cancer was lower than anticipated (*N* = 53). A statistical amendment to our analysis strategy was included as a consequence. We introduced a bootstrapping procedure. 95%CIs for the percentile range was calculated to assess precision of the two percentiles scores for both FCR4 and FCR7, by applying a bootstrap consisting of 1000 samples. The scores at each percentile level across the two sites were similar, taking into account the confidence interval for the colorectal cancer patients, that is 10 (FCR4) and 15 (FCR7) for the 60th percentile and 15 (FCR4) and 27 (FCR7) for the 90th percentile. The percent ‘zero’ scorers, that is, patients who rated themselves with the minimum score on FCR was 9% for the FCR4 and 4% for the FCR7 scale. The lower percentage of ‘zero’ scorers with the FCR7 scale is due to there being 7 opportunities rather than 4 with the FCR4 to indicate a rating on at least one item greater than the minimum level.

**Table 2:** Verbatim items including origin, frequencies, means (SDs) for breast and colorectal patient samples, with 60th and 90th percentiles, and percent ‘zero’ scorers

Question Number and Text	FCR4	FCR7	Breast (*n* = 206)	Colorectal (*n* = 53)	Total (*n* = 259)
			m (sd)	m (sd)	m (sd)
Q1: I am afraid that my cancer may recur^1^	Fcr1	Fcr1	2.55 (1.01)	2.26 (0.98)	2.49 (1.01)
Q2: I am worried or anxious about the possibility of cancer recurrence^2^	Fcr2	Fcr2	2.31 (1.00)	2.11 (1.05)	2.27 (1.01)
Q3: How often have you worried about the possibility of getting cancer again^3^	Fcr3	Fcr3	2.74 (0.90)	2.51 (1.01)	2.69 (0.93)
Q4: I get waves of strong feelings about the cancer coming back^4^	Fcr4	Fcr4	1.95 (1.08)	1.85 (1.08)	1.93 (1.08)
Q5: I think about the cancer returning when I didn’t mean to^4^		Fcr5	1.97 (0.92)	1.77 (0.85)	1.93 (0.91)
Q6: I examine myself to see if I have physical signs of cancer^2^		Fcr6	2.84 (1.04)	2.06 (1.13)	2.68 (1.10)
Q7: To what extent does worry about getting cancer again spill over or intrude on your thoughts and activities^3^		Fcr7	2.61 (2.49)	2.34 (2.41)	2.56 (2.47)
Total FCR4			9.54 (3.60)	8.74 (3.79)	9.37 (3.65)
60th percentile			10	9^a^	10
90th percentile			15	16^a^	15
Total FCR7			16.96 (6.88)	14.91 (7.33)	16.54 (7.01)
60th percentile			17	16^b^	17
90th percentile			27	28^b^	27
‘Zero’ scorers					
FCR4 percent: N			7%, 15	15%, 8	9%, 23
FCR7 percent: N			2%, 5	8%, 4	4%, 9

^a^Confidence interval around point estimate ranges 5 units (bootstrap)

^b^Confidence interval around point estimate ranges 8 units (bootstrap)

^1^From Concern about Recurrence Scale [32]

^2^From Fears of Cancer Recurrence Inventory [27]

^3^From Worry of Cancer Scale [34]

^4^From FORPSYCH study [35]

### Psychometric properties CTT: Structure and internal consistency

**Fig. 1:**
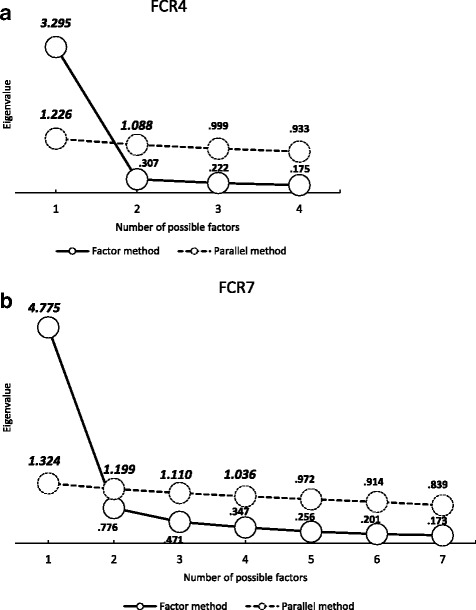
Exploratory Factor analysis using Parallel Analysis (**a**) FCR4 (**b**) FCR7

To satisfy the second aim, the investigators began by conducting exploratory factor analyses using parallel analysis to derive simulated eigenvalues from random samples for comparing with the observed data was conducted. The factor loadings for all 7 items were all high (> 0.7) with the exception of Question 6 which was 0.45. The Kaiser-Meyer-Olkin test (indicates if sufficient common variance exists to merit factor analysis) gave satisfactory high values of 0.86 and 0.92 for FRC4 and FCR7, respectively [21]. The factor analyses demonstrated single factor structures (eigenvalue for first factor = 3.3 and 4.8, for FRC4 and FCR7 respectively), which is equivalent to three and nearly 5 times the average amount of variance contained within the single factors of FCR4 and FCR7 as the rest of each of the covariance matrices. Eigenvalues derived from the bootstrapping procedure, that selects multiple random samples (*n* = 1000) from the original data set, showed that 2 and 4 factors (FCR4 and FCR7 respectively) would have been selected (eigenvalues ranging greater than 1) if the conventional, but now criticised unity criteria [23] for setting the number of factors had been recognised (Fig. 1a and b). We ran a confirmatory factor analysis to test the invariance of the unidimensional model across cancer sites and the chi square test returned a low values for FCR4 (chi-sq. = 2.97, df3, *p* = 0.39) and FCR7 (chi-sq. = 4.14, df6, *p* = 0.66) demonstrating that the measurement properties were not sufficiently different across sites to warrant a change of weighting of the items [24].

The reliability coefficients were acceptable for both scale versions (Table 3), that is above 0.9. It was interesting that even restricting the measure to the first 3 items of the FCR4 returned a Cronbach’s alpha of 0.92 for the whole sample.

**Table 3:** Psychometrics from exploratory factor analysis and reliability

		FCR4	FCR7
Factor loadings (EFA)			
	Q1	0.91	0.88
	Q2	0.93	0.91
	Q3	0.91	0.89
	Q4	0.88	0.88
	Q5	–	0.80
	Q6	–	0.52
	Q7	–	0.85
Kaiser-Meyer-Olkin Measure of Sampling Adequacy		0.859	0.920
Internal consistency			
	Cronbach’s alpha (α)	0.93	0.92
	(95%CI)	(0.91, 0.94)	(0.90, 0.94)

### Psychometric properties CTT: Validity

**Fig. 2:**
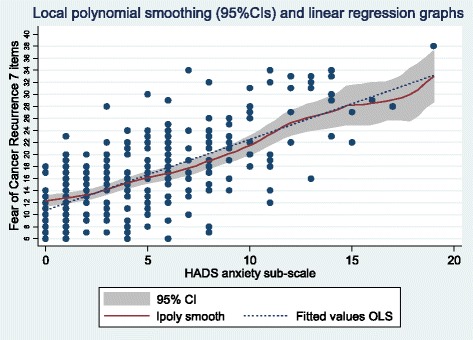
Fear of recurrence scale (7 items) and HADS Anxiety sub-scale scatterplot with polynomial smoothing and OLS linear regression line fits

Associations of FCR4 and FCR7 total scores with anxiety and depression subscales scores of the HADS were significant as calculated by Pearson’s product-moment correlation coefficient (Table 4). The comparison between the correlations of FCR4 and FCR7 with anxiety (≈ 0.66) and depression (≈ 0.38) showed that fears of recurrence are associated more strongly with anxiety than depression (difference in correlations: z ≈ 4.5, *p* < 0.0001). The scattergram of FCR7 total values with the HADS Anxiety subscale shows a close linear relationship as confirmed by the close correspondence of the polynomial smoothed line and the summary Ordinary Least Square fitted line (Fig. 2). The HADS subscale Mean (SD) score for anxiety was 4.15 (3.37) *n* = 227 for those below the cut-off (15) on the FCR4 and 10.56 (3.84) *n* = 32 at or above cut-off (*t* = 9.91, df257, *p* < .0001). The equivalent HADS Anxiety values at the cut-off (27) for FCR7 was 4.17 (3.30) *n* = 232 and 11.56 (3.60) *n* = 27 (*t* = 10.90, df257, *p* < .0001).

**Table 4:** Pearson Correlation matrices for nomological construct validity check

			Pearson Correlation Coefficients			
	mean	SD	FCR4	FCR7	HADS: Anxiety Sub-scale	HADS: Depression Sub-scale
FCR4	9.37	3.65	1	.954**	.648**	.360**
FCR7	16.54	7.01		1	.678**	.404**
HADS: Anxiety Sub-scale	4.94	4.02			1	.610**
HADS: Depression Sub-scale	2.30	2.58				1

**p < 0.01 (2-tailed); *N* = 259

**Fig. 3:**
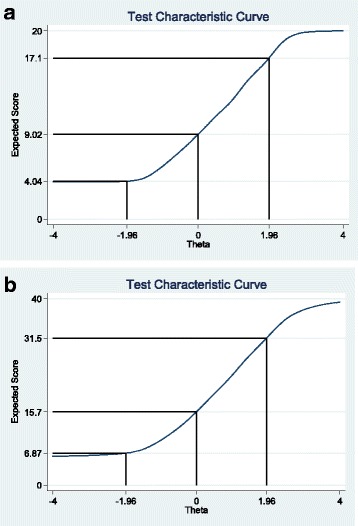
IRT Test Information Curves for total scale scores (**a**) FCR4 (**b**) FCR7 with ±1.96 Theta values marked with solid line to show the 95% proportion of sample and corresponding upper and lower bound scale

### Associations with related constructs that are central to the modified SRM of Lee-Jones et al. (1997) [15]

Spearman rho correlation coefficients between the single item questions and FCR4 and FCR7 to test construct validity all showed strong associations as predicted (Table 5). Relatively smaller coefficients were found with the coping strategy of ‘ignore feelings about the cancer returning’.

**Table 5:** Spearman Correlations of self-reported Symptoms, Coping strategies and Risk beliefs with FCR4 and FCR7

Construct validity Item		FCR4	FCR7
Symptoms			
	‘Minor aches and pains remind me of cancer’	.629*	.631
Coping Strategy			
	‘I just try to ignore feelings about the cancer returning’	.290	.283
Risk of Recurrence			
	‘In your opinion, what is your risk of having cancer recurrence?’	.569	.567

*All coefficients (Spearman’s rho) *p* < 0.001

### Influence of past treatment (Rx and CRx)

Patients who had radiotherapy in their primary treatment protocol were relatively more fearful on both measures compared with patients who did not receive radiotherapy, but these mean differences were not significant (*p* = > 0.30). Chemotherapy treatment was associated with increased FCR (*p* = 0.006) compared to those not receiving chemotherapy equivalent to approximately an effect size of 0.4 (Table 6).

**Table 6:** Past treatment history related to FCR4 and FCR7 total scores

		Radiotherapy			Chemotherapy		
		FCR4	FCR7		FCR4	FCR7	
				N			N
Yes	M	9.54	16.99	140	10.32	18.40	78
	SD	3.61	7.13		3.77	7.55	
No	M	9.19	16.05	106	8.96	15.74	168
	SD	3.72	6.95		3.53	6.67	
	F	0.57	1.09		7.60	7.75	
	*p*	0.45	0.30		0.006	0.006	

### Psychometric properties IRT

The IRT assumption of unidimensionality was tested and shown by the CFA results presented already, and also by calculating the ratio of the first to second eigenvalue being greater than 4. The ratios for the FCR4 and FCR7 were 10.7 and 6.2 respectively. The second IRT assumption of local independence of item pairs was regarded as met as each item was worded differently. Each item invited respondents to reflect different aspects of the experience of FCR. Hence, the two parameter model was applied and fitted the responses to all items relatively well as shown by the discriminability coefficients (Table 7). All coefficients for the FCR4 items were very close or above 4, that is they were above 1.7 (noted as ‘very high’ according to the criteria of Baker) [25]. The addition of three items (‘fcr5’ to ‘fcr7’) also give significant coefficient levels but the ‘fcr6’ item returned a level of 0.93, which was noted as ‘moderate’ according to the Baker criteria. The item difficulty levels (also known as severity or location levels) along the latent construct were all showing significant fit for each category of the rating scales with two exceptions in the items ‘fcr1’ and ‘fcr4’ (Table 7). The average item SEs for the difficulty (location or severity) coefficients was 0.162 (and none of the averages for each item were above recommended level of 0.35) [26]. The test characteristic curves for both versions (FCR4 and FCR7) demonstrate that 95% of the sample answer within a reasonable full range of scores (Fig. 3). That is the range of values for FCR4 is between scale scores of 4 and 17, whereas the FCR7 effective range is 7 to 32. For detailed item information the reader is invited to see the Additional file 2: IRT supplementary.

**Table 7:** IRT (2 parameter model) Summary results showing discriminability (‘discrim’) and difficulty (‘diff’, i.e. location or severity) coefficients

	Coeff.	SE	z	*P*
fcr1				
Discrim	3.99	.451	8.86	0.000
Diff				
>=2	−1.03	.108	−9.53	0.000
>=3	.05	.080	0.65	0.517
>=4	1.27	.116	10.93	0.000
=5	1.90	.154	12.38	0.000
fcr2				
Discrim	5.23	.667	7.84	0.000
Diff				
>=2	−.72	.091	−7.95	0.000
>=3	.39	.080	4.92	0.000
>=4	1.17	.108	10.82	0.000
=5	2.21	.180	12.26	0.000
fcr3				
Discrim	5.12	.689	7.43	0.000
Diff				
>=2	−1.30	.119	−10.93	0.000
>=3	−.26	.080	−3.27	0.001
>=4	1.03	.102	10.08	0.000
=5	2.07	.164	12.62	0.000
fcr4				
Discrim	4.18	.515	8.12	0.000
Diff				
>=2	−.03	.080	−0.41	0.682
>=3	.52	.086	6.09	0.000
>=4	1.45	.126	11.52	0.000
=5	2.28	.197	11.58	0.000
fcr5				
Discrim	2.47	.283	8.74	0.000
Diff				
>=2	−.27	.095	−2.87	0.004
>=3	.61	.103	5.95	0.000
>=4	2.32	.224	10.34	0.000
=5	3.36	.500	6.73	0.000
fcr6				
Discrim	.927	.141	6.56	0.000
Diff				
>=2	−1.85	.306	−6.05	0.000
>=3	−.51	.171	−2.99	0.003
>=4	1.65	.277	5.98	0.000
=5	3.47	.544	6.38	0.000
fcr7				
Discrim	2.70	.262	10.30	0.000
Diff				
>= 1	−.94	.114	−8.30	0.000
>= 2	−.22	.089	−2.51	0.012
>= 3	.20	.088	2.28	0.023
>= 4	.68	.100	6.82	0.000
>= 5	.99	.113	8.75	0.000
>= 6	1.26	.128	9.87	0.000
>= 7	1.58	.147	10.73	0.000
>= 8	1.73	.156	11.07	0.000
>= 9	2.39	.220	10.89	0.000
=10	2.68	.268	9.98	0.000

## Discussion

In this study we set out to evaluate low patient burden questionnaires to assess FCR. Some classical psychometric analyses (CTT) [27] were employed to assess the ability of the items to reflect patients’ recurrence fears in patients attending out-patient clinics having been treated for either breast or colorectal cancer. Of importance was the ability of the measures to detect ‘moderate’ to ‘high’ levels of FCR defined by the percentile points of the 60th and 90th percentiles. Percentile range for patients with colorectal cancer was wide due to small sample size, however the percentile value was well within the range for the patient counterparts with breast cancer. The percentile points (60th and 90th) were chosen from a pragmatic view to assist clinicians to identify where they might consider to invite patients to explore FCR (moderate level) or consider providing more formal support (high level). The significance of the 60th and 90th percentile is to act as indicators for clinical team members to enable them to alert themselves when patients score above these values in their outpatient clinics. We suggest that the 60th percentile would be a level that the clinical team might consider broaching the subject of FCR with the patient to provide some psycho-educational support. There were no statistical differences between item means and total scores (FCR4 and FCR7) across cancer sites. The 90th percentile might again be used as a trigger to offer not only regular support but possibly some therapeutic intervention. These scales therefore may assist clinical teams in designing programmes for stepped care for persons moving through their treatment protocols and outpatients [28].

### Psychometric properties CTT

The exploraty factor analyses on both FCR4 and FCR7 demonstrated clearly that both scales were uni-dimensional in structure as shown by the large eigenvalue for the first factor on both versions. A close inspection of the factor loadings indicates good correspondence with the latent factor. A possible exception to this was Q6 (self-examination) with a loading of 0.52. Overall we consider there is sufficient evidence from this initial analysis to support a unidimensional structure for both scales.

The non-significance result to test cancer site invariance of the factor loadings provided some limited evidence of no appreciable difference of measurement structure for both versions (FCR4 and FCR7) across cancer sites. Larger sample sizes for each cancer site would give more robust results.

The internal consistency, as assessed by Cronbach alphas, for both scales was above 0.92. The lower bound of the 95% confidence interval was reassuringly above the 0.9 level. These high values for relatively short scales are helpful to clinical teams and researchers wishing to include an assessment for FCR. The burden on patients is low, readability is acceptable, and there were no missing responses to the items and little, if any, negative reaction of patients to an invitation to complete the 7 item scale.

Further research with other samples, languages and cancer sites are recommended to replicate these results.

### Psychometric properties CTT: Validity

Associations with HADS anxiety and depression were confirmed and are consistent with previous reports in reviews and FCR surveys [1, 29].

The linear relationship with anxiety is convincing when examining the scattergram presentation and comparing the polynomial smoothed curve and the ‘ordinary least squares’ regression summary line. The levels of anxiety and depression are very similar to those reported in a mixed cancer sample (*n* = 60) from Quebec [30]. The HADS anxiety subscale scores between groups separated by the cut-offs on both FCR4 And FCR7, suggested by the ROC analyses (contact author for details), showed a greater effect size (1.87) in comparison to the Canadian study and its comparison between ‘clinical’ and ‘non-clinical’ groups (0.86).

The rank correlations between three of the fundamental constructs in the SRM of Leventhal [31], as formulated in reference to FCR [15], namely: Symptoms, Coping strategy and Risk of recurrence was reassuring. As predicted the denial of feelings strategy was the weaker of the associations presented, possibly reflecting the process of denial as a hidden activity for respondents to admit to and therefore attenuating a possible strong correlation.

### Influence of past treatment (Rx and CRx)

The small positive associations between FCR and the two principal modes of treatment (other than surgery) namely: radiotherapy and chemotherapy were detected. The association between chemotherapy and FCR was significant and matches a previous meta-analysis [19]. The measures would appear to be able to reflect reliable but small effects.

### Psychometric properties IRT

The Item Response Theory results concentrated on each of the items that compose the FCR4 and FCR7 scales in detail. The discrimination and difficulty levels across the items and categories were virtually all statistically significant. Of interest is the strength of discriminability of the FCR4 items which all attained a substantial level of 4. The other three items that are added to FCR7 do not attain such high levels but it can be argued that the longer scale is attempting to assess a broader concept of FCR. The advantage of the additional three items to the FCR4 is shown in the wider coverage of difficulty (or location) that the 7 items provide (Category Characteristic Curves in Additional file 2, especially for item ‘fcrq6’). This is one of the first presentations of FCR short measures adopting a two parameter IRT model. We argue that there is good evidence to support the assumption of unidimensionality from the EFA, however the local independence assumption is a matter of judgement from assessing the uniqueness of the item wording. This aspect may require additional attention in future studies.

### Choice of version: FCR4 or FCR7?

The selection of FCR assessment, whether focussing on these brief measures or others, is dependent on a variety of factors. These will include preference for wording, time allocated for patient profiling and purpose of study or service function. From a psychometric perspective there is little to choose between the FCR4 and FCR7. If the interest of the user is to identify patients, say for research purposes, who report no recurrence fears (so called ‘zero’-scorers) then the FCR7 is again the preference as the respondent has multiple opportunities to confirm that they are not concerned. A mobile phone application has been developed for routine use for participants involved in research studies. Recommended guidelines were adhered to [32]. If the user wishes to assess repeatedly over a relatively short duration then the FCR4 would be indicated as the respondent burden is less. There is no meaningful difference in the reliability coefficients’ magnitude between the 2 versions. In addition, classical validity evidence that has so far been presented is consistent across versions.

The IRT evidence shows an interesting phenomenon. The additional information provided by the technique highlights that the FCR7, in addition to assessing aspects of fear and anxiety as the FCR4 does also includes two more cognitive aspects (i.e. intrusions) and a coping strategy (behavioural checking). A recent comment has highlighted that some assessments of FCR are clearly designed to assess intensity of fear such as the FCRI-Severity subscale (9 items), whereas the CAR-Q includes 3 aspects of clinical symptoms that Lebel et al. [33] have argued are indicative of clinical levels of FCR [34]. Our position would be that the FCR4 is a quick screen for fear itself of FCR, whereas the FCR7 would function as a possible indicator of 3 of the clinical issues recommended for assessment that could trigger early intervention [35]. We would encourage additional item development if the researcher / clinician wished to adopt a measure to locate clinical FCR. The 42 item Fear of Cancer Recurrence Inventory (FCRI) would be a suitable assessment for individuals to complete should their score attain a level at the 90th percentile or higher. It is noted that this has already been translated successfully into a number of major languages [5, 36, 37].

### Limitations and strengths

The assessment of FCR has been clarified with a consensus definition now available [33]. These two brief scales are limited to recurrence and are not suitable to assess fear of progression of cancer. We are currently developing a parallel version for the additional component described in the definition. Sample size, in the current study, for the colorectal cancer patients was low, however their inclusion provided some evidence that the discrepancy in the measures across the two sites was not appreciative, supporting the view that separate FCR scales for each cancer site is unwarranted. Further structural testing is required. The sample of patients collected with breast and colorectal cancer was not necessarily representative of patients with these two conditions due to patients being withdrawn by their clinical teams for further medical investigations. However the efficient use of IRT evaluation has been described with a small clinical sample comparing three common depression instruments [38]. These brief measures we believe are faithful to the position adopted by Costa who recommended clear and brief instrumentation for FCR [6]. The FCR4 and FCR7 are measures comprising items from four other FCR measures. Therefore the user of the FCR4 or FCR7 will be able to make reference of discrete items directly to the other studies of recurrence fears who report details of the identical items.

Two further issues are raised for consideration. Psychometric developments in assessing FCR are encouraging and becoming more sophisticated. The use of alternative models such as IRT (as opposed to classical psychometric theory [27]) hold promise to further improve our understanding of the constructed assessments. There are some notable examples of IRT applied to FCR measures [7, 39]. Assumptions applied to IRT models tend to be more demanding and require large samples to derive precision of estimates, especially when random mixed models are applied. The ‘high N’ requirement would also apply to structural equation models (SEM). The SEM approach is able to explicitly test assumptions of measurement principles, within the items that compose the scale (e.g. correlated residuals), as well as construct validation with detailed examination of associated constructs (e.g. cross-construct loadings). As raised previously Simonelli criticises the preponderance of single point investigations and lack of cross-cultural validity [3]. We would recommend that investigators consider how changes in FCR might be assessed in addition to concentration on the ‘steady-state’ FCR estimation. Finally we are aware that some patients rate their FCR as effectively zero. The construct of FCR is conceptually different to either anxiety or depression. The more traditional assessments of negative mental state concentrate on the higher levels. For FCR this would not be the case. Patients who have zero scores on FCR have either misunderstood the measure’s instructions and rating scheme, avoiding exposure to rating the construct itself (through denial for example) or are genuinely unfearful of the possibility of cancer return. The consequences of the ‘zero-scorer’ are unknown in terms of health behaviour, out-patients visits or self-checking for a recurrence.

## Conclusion

The FCR4 and FCR7 are brief measures of FCR that are designed to be easy, low burden scales for use in clinical settings and research enquiries. Some psychometric qualities are supportive of their internal consistency and evidence for validity. Additional research to extend the background information about these scales to assist interpretation are required and warrant investigation.

## Additional files

Additional file 1: FCR7 for copy of Scale. (DOCX 114 kb)

Additional file 2: IRT Supplementary. (DOCX 72 kb)

## Abbreviations

AUC: Area Under Curve
CAR-Q: Concerns About Recurrence Questionnaire
CCT: Classical Test Theory
FCR4: Fears of Cancer Recurrence - 4 Item Version
FCR7: Fears of Cancer Recurrence - 7 Item Version
HADS: Hospital Anxiety and Depression Scale
IRT: Item Response Theory
SRM: Self Regulation Model
UoWQoL: University of Washington Quality of Life
