# Supplementary material for: Unidimensional scales for fears of cancer recurrence and their psychometric properties: the FCR4 and FCR7
Source: Health Qual Life Outcomes. 2018 Feb 9;16:30. doi: 10.1186/s12955-018-0850-x (PMC5822647; doi:10.1186/s12955-018-0850-x)
Supplement: Supplementary file 1 — FCR7 for copy of Scale. (DOCX 114 kb) [file 12955_2018_850_MOESM1_ESM.docx]

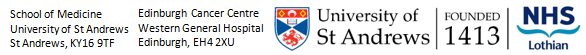


Name

Date

Patient identification number*: [*For Office Use]
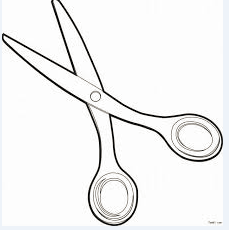


**FCR7 Items^†^**

Please answer the following questions by placing a tick in any of the boxes for each of the question.

You do not have to answer these questions if you do not wish. We will anonymise your information.

Please hand this scale to your specialist. Thank you!

|  | Not at all | | A little | | Sometimes | | | A lot | | All the time | | |
| --- | --- | --- | --- | --- | --- | --- | --- | --- | --- | --- | --- | --- |
|  | **1** | | **2** | | **3** | | | **4** | | **5** | | |
| Q1: I am afraid that my cancer may recur | □ | | □ | | □ | | | □ | | □ | | |
| Q2: I am worried or anxious about the possibility of cancer recurrence | □ | | □ | | □ | | | □ | | □ | | |
| Q3: How often have you worried about the possibility of getting cancer again | □ | | □ | | □ | | | □ | | □ | | |
| Q4: I get waves of strong feelings about the cancer coming back | □ | | □ | | □ | | | □ | | □ | | |
| Q5: I think about the cancer returning when I didn’t mean to | □ | | □ | | □ | | | □ | | □ | | |
| Q6: I examine myself to see if I have physical signs of cancer | □ | | □ | | □ | | | □ | | □ | | |
|  | **Not at all** | | | |  | | | **A great deal** | | | | |
| Q7: To what extent does worry about getting cancer again spill over or intrude  on your thoughts and activities | **0** | **1** | **2** | **3** | **4** | **5** | **6** | **7** | **8** | | **9** | **10** |

**Thank you for your support!**

^† FCR4 is sum of first 4 items (Q1 to Q4)^
