# Supplementary material for: Unidimensional scales for fears of cancer recurrence and their psychometric properties: the FCR4 and FCR7
Source: Health Qual Life Outcomes. 2018 Feb 9;16:30. doi: 10.1186/s12955-018-0850-x (PMC5822647; doi:10.1186/s12955-018-0850-x)
Supplement: Supplementary file 2 — IRT Supplementary. (DOCX 72 kb) [file 12955_2018_850_MOESM2_ESM.docx]

Items fcrq1 to fcrq7 (CC Curves)

Items fcrq1 to fcrq7

Scales FCR4 and FCR7 Information Functions
